# Burdett's Hospitals and Charities

**Published:** 1900-08-25

**Authors:** 


### EDITOR'S LETTER-BOX.

[Our Correspondents are reminded that prolixity is a great bar to publication, and that brevity of style and conciseness of statement greatly facilitate early insertion.]

### "BURDETT'S HOSPITALS AND CHARITIES."

Mr. J. G. Craggs writes: May I criticise a critic? There is in your issue of July 21st last a review of "Burdett's Hospitals and Charities, 1900," in which the reviewer expresses himself as follows:—

"We are inclined to think that some of the institutions given are not altogether worthy of their position in this volume. Thus, some hospitals have not responded to the request for details of their constitution, accommodation, &c., for several years. Might not such institutions be omitted? It seems a pity to occupy valuable space with information which may no longer be accurate."

I take an entirely opposite view to that of the reviewer, and I think the inclusion of the institutions in question is most valuable and entirely justifies the space they occupy, provided they are (as is the case) carefully marked to show that they have not responded to the request for details, accounts, &c. Your reviewer appears to have quite overlooked what I believe to be the only sound principle in charitable undertakings, viz., that whatever institution appeals to the public for support must, in return, fearlessly publish the fullest information respecting both its work and accounts. Consequently, it is the bounden duty of all responsible persons to facilitate the work of an author of such a book as "Burdett's Hospitals and Charities," in placing at everybody's service all necessary details to enable anyone by an immediate reference to form an opinion of the *bonâ fides* of any given institution. I have always taken an interest in hospitals, and especially so during the past few years as honorary secretary of the Prince of Wales's Hospital Fund for London. I therefore feel it my duty to write to you upon this point, but it is as a chartered accountant that I feel that my opinion is of some value. The public are put upon their guard if they see any institution specially marked, and are warned that they should make further inquiries in any such case. Publicity is practically the chief guard against many easy forms of fraud. I know a friend who, as a chartered accountant, has recently been investigating a serious fraud spread over several years, and I feel sure that upon reference it will be found that this institution must have been "starred" in Sir Henry Burdett's valuable work of reference.

*** We agree with Mr. Craggs' contention in the above letter. To show the utility of the asterisk we may refer to the announcement in our issue of August 4th of the withdrawal of Royal patronage from the Royal Military Benevolent Fund, 193, Queen's Gate, which, as we understand, resulted from the presence of this asterisk opposite the entry in "Burdett's Hospitals and Charities" having led to inquiries being made as to the management of the institution.

